# Kinetics of HIV-1 CTL Epitopes Recognized by HLA I Alleles in HIV-Infected Individuals at Times near Primary Infection: The Provir/Latitude45 Study

**DOI:** 10.1371/journal.pone.0100452

**Published:** 2014-06-25

**Authors:** Jennifer Papuchon, Patricia Pinson, Gwenda-Line Guidicelli, Pantxika Bellecave, Réjean Thomas, Roger LeBlanc, Sandrine Reigadas, Jean-Luc Taupin, Jean Guy Baril, Jean Pierre Routy, Mark Wainberg, Hervé Fleury, the Provir/Latitude 45 study group

**Affiliations:** 1 University Hospital of Bordeaux and CNRS UMR 5234, Bordeaux, France; 2 University Hospital of Bordeaux and CNRS UMR 5164, Bordeaux, France; 3 Clinique médicale l'Actuel, Montréal, Canada; 4 Clinique médicale Opus, Montréal, Canada; 5 Clinique Médicale du Quartier Latin, Montréal, Canada; 6 McGill University Health Centre, Montréal, Canada; 7 McGill AIDS Centre, Montréal, Canada; University of Cape Town, South Africa

## Abstract

In patients responding successfully to ART, the next therapeutic step is viral cure. An interesting strategy is antiviral vaccination, particularly involving CD8 T cell epitopes. However, attempts at vaccination are dependent on the immunogenetic background of individuals. The Provir/Latitude 45 project aims to investigate which CTL epitopes in proviral HIV-1 will be recognized by the immune system when HLA alleles are taken into consideration. A prior study (Papuchon et al, PLoS ONE 2013) showed that chronically-infected patients under successful ART exhibited variations of proviral CTL epitopes compared to a reference viral strain (HXB2) and that a generic vaccine may not be efficient. Here, we investigated viral and/or proviral CTL epitopes at different time points in recently infected individuals of the Canadian primary HIV infection cohort and assessed the affinity of these epitopes for HLA alleles during the study period. An analysis of the results confirms that it is not possible to fully predict which epitopes will be recognized by the HLA alleles of the patients if the reference sequences and epitopes are taken as the basis of simulation. Epitopes may be seen to vary in circulating RNA and proviral DNA. Despite this confirmation, the overall variability of the epitopes was low in these patients who are temporally close to primary infection.

## Introduction

HIV-1 infection is a chronic infection with non-stop viral replication leading to a decrease in the number of T CD4 lymphocytes and immunosuppression. Viral replication can be limited by antiretroviral drugs of different classes. This reduction in viral replication, which is generally below the threshold of the limit of detectability of viral load (VL) commercial assays, is followed by an increase in numbers of T CD4 lymphocytes. However, antiretroviral treatment (ART) cannot be stopped even in fully responding patients, since various clinical trials have shown that its interruption is followed by the resumption of viral replication [1]. The viral reservoirs include gut-associated lymphoid tissues (GALT), the central nervous system, the genital tract and the lymph nodes, which are not fully permeable to ARV. The role of long-term resting memory T CD4 lymphocytes as cellular reservoir of latent but replication-competent virus is now understood [2].

Whether viral replication under the VL threshold occurs in patients at full success of ART is still a matter of debate. Some groups consider that low level viral replication might replenish the viral reservoir [3]–[4]. The picture is more complicated than thought depending on the tools used to assess the viral latent reservoir and whether the defective or competent viruses are taken into account or not [5]. In patients responding successfully to ART, the next step is viral cure. Among attempts at viral cure [6], antiviral vaccination, particularly involving T CD8 epitopes, is a promising strategy since the importance of the T CD8 cytotoxic response in the decrease in viral replication during the primary infection phase of the disease is well known [7]–[9]. These epitopes are HLA-restricted and must be presented by molecules encoded by HLA alleles (mainly A and B), therefore introducing a genetic and individual parameter into the pathophysiology of the immune response and the evolution of the disease [10]–[15]. However, the curative vaccines used so far have been based on CTL epitopes from generic reference circulating HIV-1 strains. Assuming that the resuming viral replication originates from proviral DNA, the question is whether or not the corresponding CTL epitopes are similar to those used for vaccination and whether they can be efficiently presented by the HLA alleles I. In a previous study [16], we showed that it is not possible to predict which CTL epitopes are archived in the proviral DNA or which will be recognized by the immune system. Therefore, a question remains as to the efficiency of generic vaccines for the control of emerging virus. While our previous study was devoted to patients at success of first line ART but far from primary infection, the present study addressed the question of the variability and potential recognition of CTL epitopes in patients close to primary infection. We therefore sequenced Gag plus RT and Nef parts of HIV-1(regions known for harboring numerous important CTL epitopes) in different samples (viral RNA and/or proviral DNA) and their potential theoretical recognition by the HLA alleles of patients. Some of the patients were treated with success during the study period so we focused on the proviral DNA obtained under success, assuming that a curative vaccination would be interesting at that step and that the vaccine epitopes, of either recombinant vaccine or lipopeptide origin, could be expected to be similar to those archived and to stimulate an efficient CTL response.

## Results

Six patients from the Canadian primary infection cohort were studied at different time points after primary infection (Table 1), an event that had occurred between 2.5 and 4.5 months before recruitment. All were Caucasian MSM infected with subtype B HIV-1. The samples investigated during the follow-up were viral RNA and/or proviral DNA; T CD4 counts ranged from 208 to 1680/uL at entry (with 3 out of 6>500/uL); viral load (VL) ranged from 9,861 copies/mL to 192,412 copies/mL. Four patients received an ART during the study period and were therefore able to provide samples (proviral DNA) while under successful treatment (with a VL at 1.6 log).

**Table 1:** Patients' characteristics.

Patient	HLA	Follow-up after primary infection				
GOL040	A*02:01	Duration after primary infection (months)	2.5	3.5	6.5	8.5
	A*24:02	Viral load (cp/mL)	189343	173044	81037	40
	B*08:01	CD4 (cells/μL)	407	430	396	567
	B*18:01	cART	none	none	none	FTC+TDF+ATV/r
CQLMAC03010	A*01:01	Duration after primary infection (months)	3.5	7.5	12.5	
	A*68:02	Viral load (copies/mL)	74293	24529	ND	
	B*14:02	CD4 T cells (cells/μL)	580	610	ND	
	B*07:02	cART	none	none	none	
ACT 87524	A*02:01	Duration after primary infection (months)	3.5	10	34	
	A*33:03	Viral load (copies/mL)	70316	45268	40	
	B*50:01	CD4 T cells (cells/μL)	620	480	950	
	B*58:01	cART	none	none	FTC+TDF+ATV/r	
HTM 399	A*01:01	Duration after primary infection (months)	4	16	28	
	A*01:01	Viral load (copies/mL)	192412	40	40	
	B*35:01	CD4 T cells (cells/μL)	208	477	716	
	B*51:01	cART	none	FTC+TDF+ATV/r	FTC+TDF+ATV/r	
GOL041	A*02:01	Duration after primary infection (months)	4.5	5	9	22
	A*29:02	Viral load (copies/mL)	9861	8042	27536	40
	B*08:01	CD4 T cells (cells/μL)	512	398	281	604
	B*44:03	cART	none	none	none	FTC+TDF+ATV/r
ACT 95387	A*03:01	Duration after primary infection (months)	4.5	5.5	17.5	29.5
	A*30:02	Viral load (copies/mL)	84725	2189	88741	34352
	B*07:02	CD4 T cells (cells/μL)	1680	1670	650	610
	B*18:01	cART	none	none	none	none

Legend to Table 2 to 7B: The CTL epitopes that should be recognized according to the HLA alleles are defined using the HXB2 reference. The observed corresponding epitopes in the different samples are described either for RNA or DNA. The dates are the dates of sampling.

« Id » indicates that the epitope observed is identical to the HxB2 reference. The numbers in parenthesis are the theorical MHC IC_50_ (nM) according to simulation of affinity between the epitopes and the HLA alleles. The numbers in parenthesis and italic represent percentages of RT variants determined by UDPS.

After molecular characterization of HLA alleles A and B, we carried out sequencing of Gag and Nef by the Sanger method while RT was investigated by a next generation sequencing method (UDPS 454 technology). According to the HLA A and B alleles, the CTL epitopes that could be presented were identified by using the immune epitope database (iedb) simulator. The number of CTL epitopes that could be studied on the basis of the sequences obtained (Gag, Nef and RT) and according to the HLA alleles ranged from 9 to 19 per patient. We decided to focus only on those epitopes that are theoretically well presented by the corresponding HLA alleles, i.e. with a theoretical value of MHC IC_50_<500 nM. Therefore, these epitopes were analyzed at different time points, provided that the corresponding sequences could be obtained.

The corresponding data are presented in Tables 2 to 7

**Table 2:** Patient GOL040.

HLA	Epitope location	HXB2 epitope sequence (MHC IC_50_)	Sampling dates after primary infection (*% of RT UDPS variants)*						
			2.5 months	3.5 months	3.5 months	6.5 months	6.5 months		8.5 months
			RNA	RNA	DNA	RNA	DNA	treatment	DNA
A*02:01	p17	SLYNTVATL	Id	Id	Id	Id	Id		Id
	(77–85)	(256.45)							
	RT	ALVEICTEM	ALVEICTEM	ALVEICTEM	ALVEICTEM	ALVEICTEM	ALVEICTEM		ALVEICTEM
	(33–41)	(41.72)	(*50%*)	(*100%*)	(*100%*)	(*100%*)	(*100%*)		*(90%)*
			(41.72)	(41.72)	(41.72)	(41.72)	(41.72)		(41.72)
			ALIEICTEM						AIIEICTEM
			(*50%*)						(*10%*)
			(15.61)						(215.37)
B*08:01	p17	ELRSLYNTV	EFRSLYNTV	EFRSLYNTV	EFRSLYNTV	EFRSLYNTV	EFRSLYNTV		EFRSLYNTV
	(74–82)	(359.95)	(2264.98)	(2264.98)	(2264.98)	(2264.98)	(2264.98)		(2264.98)
	p24	EIYKRWII	Id	Id	Id	Id	Id		Id
	(128–135)	(257.38)							
	RT	GPKVKQWPL	GPKVKQWPL	GPKVKQWPL	GPKVKQWPL	GPKVKQWPL	GPKVKQWPL		GPKVKQWPL
	(18–26)	(72.58)	(*100%*)	(*100%*)	(*100%*)	(*100%*)	(*100%*)		(*100%*)
			(72.58)	(72.58)	(72.58)	(72.58)	(72.58)		(72.58)
	Nef	FLKEKGGL	Id	Id	Id	Id	Id		Id
	(90–97)	(228.50)							

**Table 3:** Patient CQLMAC03010.

HLA	Epitope location	HXB2 epitope sequence (MHC IC_50_)	Sampling dates after primary infection			
			3.5 months	7.5 months	12.5 months	12.5 months
			RNA	RNA	RNA	DNA
B*07:02	p24	SPRTLNAWV	Id	Id	Id	Id
	(16–24)	(25.64)				
	p24	GPGHKARVL	Id	Id	Id	Id
	(223–231)	(97.86)				
	Nef	FPVTPQVPL	FPVRPQVPL	FPVRPQVPL	FPVRPQVPL	FPVRPQVPL
	(68–76)	(11.70)	(7.48)	(7.48)	(7.48)	(7.48)
	Nef	TPQVPLRPM	RPQVPLRPM	RPQVPLRPM	RPQVPLRPM	RPQVPLRPM
	(71–79)	(12.45)	(3.68)	(3.68)	(3.68)	(3.68)
	Nef	RPMTYKAAL	RPMTFKAAR	RPMTYKAAM	RPMTYKAAR	RPMTYKAAR
	(77–85)	(2.67)	(1544.70)	(2.71)	(1446.12)	(1446.12)

**Table 4:** Patient ACT 87524.

HLA	Epitope location	HXB2 epitope sequence (MHC IC_50_)	Sampling dates after primary infection (*% of RT UDPS variants)*				
			3.5 months	10 months	10 months		34 months
			RNA	RNA	DNA	treatment	DNA
A*02:01	p17	SLYNTVATL	SLYNTVAVL	SLYNTVAVL	SLYNTVAVL		SLYNTVAVL
	(77–85)	(256.45)	(379.80)	(379.80)	(379.80)		(379.80)
	RT	ALVEICTEM	ALVEICTEM	ALVEICTEM	ALVEICTEM		ALVEICTEM
	(33–41)	(41.72)	(*100%*)	(*100%*)	(*92%*) (41.72)		(*100%*)
			(41.72)	(41.72)	VLVEICTEM		(41.72)
					(*4%*) (66.92)		
					ALVKICTEI		
					(*4%*) (109.35)		
B*58:01	p24	TSTLQEQIGW	ND	TSNLQEQIGW	TS[NT]LQEQI[GA]W		TS[NT]LQEQI[GA]W
	(108–117)	(50.78)		(32.78)	(32.13 to 50.78)		(32.13 to 50.78)

**Table 5:** Patient HTM 399.

HLA	Epitope location	HXB2 epitope sequence (MHC IC_50_)	Sampling dates after primary infection (*% of RT UDPS variants)*			
			4 months		16 months	28 months
			RNA	treatment	DNA	DNA
B*35:01	RT	TVLDVGDAY	TVLDVGDAY		TVLDVGDAY	TVLDVGDAY
	(107–115)	(17.80)	(*100%*)		(*100%*)	(*90%*) (17.80)
			(17.80)		(17.80)	TVLDVGDVY
						(*10%*) (48.32)
	RT	VPLDEDFRKY	VPLDEDFRKY		VPLDEDFRKY	VPLDEDFRKY
	(118–127)	(67.60)	(*85%*) (67.60)		(*73%*) (67.60)	(*100%*) (67.60)
			VPLDKDFRKY		VPLDEDFRKC	
			(*5%*) (168.14)		(*27%*) (16764.23)	
			VPLDKEFRKY			
			(*10%*) (270.24)			
	RT	NPDIVIYQY	NPDIVIYQY		NPDIVIYQY	NPDIVIYQY
	(175–183)	(30.19)	(*60%*) (30.19)		(*100%*)	(*89%*)
			NPEIVIYQY		(30.19)	(30.19)
			(*30%*) (58.88)			KPDIVIYQY
			NPSIIIYQY			(*11%*)
			(*5%*) (15.58)			(200.84)
			NPEIIIYQY			
			(*5%*) (62.86)			
	Nef	VPLRPMTY	Id		Id	Id
	(74–81)	(44.34)				

**Table 6:** Patient GOL041.

HLA	Epitope location	HXB2 epitope sequence (MHC IC_50_)	Sampling dates after primary infection (*% of RT UDPS variants)*					
			4.5 months	5 months	9 months	9 months		22 months
			RNA	DNA	RNA	DNA	treatment	DNA
A*02:01	RT	ALVEICTEM	ALVEICTEM	ALVEICTEM	ALVEICTEM	ALVEICTEM		ALVEICTEM
	(33–41)	(41.72)	(*100%*)	(*100%*)	(8*8%*) (41.72)	(*100%*)		(99*%*)
			(41.72)	(41.72)	ALVKICTEM	(41.72)		(41.72)
					(*6%*) (507.81)			ALVEVCTEM
					ALMEICTEM			(1*%*)
					(*6%*) (7.25)			(41.41)
B*08:01	RT	GPKVKQWPL	GPKVKQWPL	GPKVKQWPL	GPKVKQWPL	GPKVKQWPL		GPKVKQWPL
	(18–26)	(72.58)	(*100%*)	(*100%*)	(*100%*)	(*98.8%*)		(*97.8%*) (72.58)
			(72.58)	(72.58)	(72.58)	(72.58)		GPKVKQWPS
						GPKVKQRPL		(*1.1%*) (3646.02)
						(*1.2%*)		GPKAKQWPL
						(172.47)		(*1.1%*) (80.87)

**Table 7:** Patient ACT 95387.

HLA	Epitope location	HXB2 epitope sequence (MHC IC_50_)	Sampling dates after primary infection (*% of RT UDPS variants)*				
			4.5 months	5.5 months	5.5 months	17.5 months	29.5 months
			RNA	RNA	DNA	RNA	RNA
A*03:01	p17	KIRLRPGGK	Id	Id	Id	Id	Id
	(18–26)	(181.36)					
	p17	RLRPGGKKK	Id	Id	Id	RLRPGGKKQ	RLRPGGKKQ
	(20–28)	(212.83)				(17483.36)	(17483.36)
	RT	ALVEICTEMEK	ALVEICTEMEK	ALVEICTEMEK	ALVEICTEMEK	ALVEICTEMEK	ALVEICTEMEK
	(33–43)	(313.24)	(*67%*) (313.24)	(*100%*)	(*97%*)	(*100%*)	(*96%*)
			ALTEICTEMEK	(313.24)	(313.24)	(313.24)	(313.24)
			(*25%*) (351.63)		KLVEICTEIER		ALVEICTEMEE
			ALIEICTEMEK		(*3%*)		(*4%*)
			(*8%*) (165.44)		(2238.39)		(32546.12)
	RT	KLVDFRELNK	KLVDFRELNK	ND	KLVDFRELNK	KLVDFRELNK	KLVDFRELNK
	(73–82)	(36.70)	(*100%*)		(*80%*) (36.70)	(*96%*) (36.70)	(*100%*)
			(36.70)		KLVDFKELNK	KLVDLGELNK	(36.70)
					(*20%*) (64.06)	(*4%*) (80.05)	
	RT	GIPHPAGLK	GIPHPAGLK	ND	GIPHPAGLK	GIPHPAGLK	GIPHPAGLK
	(93–101)	(316.17)	(*100%*)		(*100%*)	(*100%*)	(*96%*) (316.17)
			(316.17)		(316.17)	(316.17)	GIPHLAGLK
							(*4%*) (556.37)
	RT	AIFQSSMTK	AIFQSSMTK	AIFQSSMTK	AIFQSSMTK	AIFQSSMTK	AIFQSSMTK
	(158–166)	(12.74)	(*64%*) (12.74)	(*100%*)	(*96%*)	(*97%*)	(*99%*)
			AIFQASMTK	(12.74)	(12.74)	(12.74)	(12.74)
			(*23%*) (20.85)		AIFQSNMTK	AIYQSSMTK	AIFQINMTK
			AIFQCSMTK		(*4%*)	(3*%*)	(1*%*)
			(*7%*) (13.50)		(18.21)	(9.20)	(21.64)
			AIFQGSMTK				
			(*2.5%*) (18.30)				
			AIFQTSMTK				
			(*2.5%*) (20.04)				
	NEF	AVDLSHFLK	ALDLSHFLR	ALDLSHFLR	ALDLSHFLR	ALDLSHFLR	ALDLSHFLR
	(66–97)	(245.36)	(1944.13)	(1944.13)	(1944.13)	(1944.13)	(1944.13)
A*30:02	p17	RSLYNTVATLY	KSLYNTVATLY	KSLYNTVATLY	KSLYNTVATLY	KSLYNTVATLY	KSLYNTVATLY
	(76–86)	(17.71)	(18.12)	(18.12)	(18.12)	(18.12)	(18.12)
	RT	KQNPDIVIY	KQNPDIVIY	ND	KQNPDIVIY	KQNPDIVIY	KQNPDIVIY
	(173–181)	(56.89)	(*12%*) (56.89)		(4*%*)	(60*%*)	(60*%*)
			KQHPDIVIY		(56.89)	(56.89)	(56.89)
			(*45%*) (70.32)		KQYPDIVIY	KQHPDIVIY	KQHPDIVIY
			KQNPDIDIY		(*56%*)	(40*%*)	(40*%*)
			(*26%*) (74.12)		(77.92)	(70.32)	(70.32)
			KQYPNIVIY		KQHPDIVIY		
			(*8%*) (57.29)		(*40%*)		
			KQYPDIVIY		(70.32)		
			(*5%*) (77.92)				
			KQNQDIVIY				
			(*3%*) (74.97)				
B*07:02	p24	SPRTLNAWV	SPRTFNAWV	Id	Id	Id	Id
	(16–24)	(25.64)	(28.93)				
	p24	GPGHKARVL	Id	Id	Id	Id	Id
	(223–231)	(97.86)					
	NEF	FPVTPQVPL	FPVRPQVPL	FPVRPQVPL	FPVRPQVPL	FPVRPQVPL	FPVRPQVPL
	(68–76)	(11.70)	(7.48)	(7.48)	(7.48)	(7.48)	(7.48)
	NEF	TPQVPLRPM	RPQVPLRPM	RPQVPLRPM	RPQVPLRPM	RPQVPLRPM	RPQVPLRPM
	(71–79)	(12.45)	(3.68)	(3.68)	(3.68)	(3.68)	(3.68)
	NEF	RPMTYKAAL	RPMTFKGAL	RPMTFKGAL	RPMTFKGAL	RPMTFKGAL	RPMTFKGAL
	(77–85)	(2.67)	(2.51)	(2.51)	(2.51)	(2.51)	(2.51)

### Patient GOL 040

(Table 2) exhibited two HLA alleles A*02∶01 and B*08∶01 allowing recognition of 6 epitopes in Gag, RT and Nef at the first point (RNA sample obtained 2.5 months after viral entry); 3 out of 6 epitopes that were analyzed by Sanger did not show any residue variation inducing a MHC IC_50_>500 nM during follow-up of 5 samples, including 2 RNA points and 3 proviral DNA points (2 of which were concomitant with 2 RNA points). One epitope, p17 74–82, was never efficiently recognized in RNA and DNA samples (MHC 2264.98 nM); RT 18–26 and RT 33–41 could be investigated by UDPS: RT 18–26 exhibited no variation with 100% of the viral population being identical and well recognized; RT 33–41 showed two sub-populations at 2.5 and 8.5 months but the variants within these sub-populations maintained their theoretical affinity for the HLA groove. At the DNA point of 8.5 months, which can be considered as an archived virus at full success of ART and could constitute the target of a curative vaccine, all epitopes but one were still able to be presented, including the variants of RT epitopes.

### Patient CQLMAC03010

(Table 3) was followed at 3 RNA points and one DNA point over a 12.5-month period following the entry of the virus, the last time point being proviral DNA. With HLA B*07∶02, this patient's immune system could recognize 5 epitopes, 4 of them during the study period (although they exhibited some residue variations that did not alter theoretical affinity), while one (Nef 77–85) was less efficiently presented at most of the time points including the last one.

### Patient ACT 87524

(Table 4) donated samples at 3 time points after infection: 3.5 months (RNA), 10 months (RNA plus DNA) and 34 months (DNA). With alleles A*02∶01 and B*58∶01, 3 peptides should be efficiently presented: one (RT 33–41) which was initially well recognized showed variability in the proviral DNA at 10 months with three sub-populations that retained their affinity for the HLA groove. At the DNA point under treatment, the viral population was again homogeneous with a good theoretical presentation. The epitope p24 108–117 was investigated only by Sanger and showed double populations at 10 and 34 months without dramatic consequences on affinity for the HLA groove. 34 months post-infection at the point of treatment success, all epitopes were still recognized despite some variability.

### Patient HTM 399 (Table 5)

Had one RNA sample available for study at 4 months after infection and 2 DNA samples while he was being treated. Since T CD4 count was low at entry (208/uL), he was treated (Truvada/Ritonavir/Reyataz) as soon as HIV-1 infection was discovered. With B*35∶01, 4 peptides were presentable by the HLA groove. Nef 74–81 investigated by Sanger was stable throughout the survey period. RT 107–115 was homogeneous at 4 and 16 months post-infection but exhibited double populations at 16 months while he was receiving a successful ART. Both sub-populations were still recognized by the HLA allele B*35∶01. RT 175–183 showed four sub-populations at 4 months post-infection and two at 28 months. All variants remained under the threshold of 500 nM considering MHC IC_50_. RT 118–127 exhibited sub-populations at 4 and 16 months with a variant accounting for 27 % of the viral population at 16 months and showing no affinity for the HLA allele (MHC IC_50_ 16,764.23 nM). At the second DNA point under treatment 28 months after infection, the epitope 118–127 was again homogeneous and presented affinity for the groove.

### Patient GOL 41

(Table 6) was infected by a subtype B virus, but we were unable to amplify allparts of the genome (particularly when investigating DNA samples) and only obtained partial results. Two epitopes that should be presentable were followed throughout the study period. Epitope RT 33–41 showed variability in circulating RNA at month 9 with 6% variants exhibiting a clearly lower affinity. At 22 months, both subpopulations were able to be presented by the A*02∶01 allele. RT 18–26 was homogeneous till month 9 and exhibited three subpopulations at month 22 while the patient was under ART, with only 1.1% variant scoring at 3646.02 nM for MHC IC_50_.

### Patient ACT 95387 (Tables 7A and 7B)

Was recruited 4.5 months after primary infection. T CD4 count was high throughout the study period without any ART. Four RNA samples and one DNA sample were investigated. The last sample studied was an RNA point at 29.5 months post-infection. With HLA A*03∶01, A*30∶02 and B*07∶02, 14 peptides in different parts of the viral genome were expected to be presented. Epitope p17 18–26 was unchanged throughout the survey, while p17 20–28 exhibited a variant by Sanger at 17.5 and 29.5 months with a loss of affinity for the groove. Nef 66–97 was not recognized whatever was the point considered, while p17 76–86 showed a residue substitution but without any significant consequence on presentation. All epitopes presented by B*07:02 were identical or very similar to the theoretical epitopes and continued to be recognized. RT 73–82, RT 158–166 and RT 173–181 exhibited sub-populations but kept their affinity at all points. Sub-populations were recognized by UDPS for RT 33–43 with low percentages of variants (3% and 4% respectively) at RNA points 5.5 and 29.5 months post-infection exhibiting little or no affinity for the HLA groove. RT 93–101 was homogeneous till the RNA point 29.5 months post-infection with 4% of a variant and an MHC IC_50_ above the threshold.

## Discussion

In our earlier study [16], we investigated patients experiencing success at first-line ART and who began therapy long - several years for most - after their primary infection. Considering the HLA alleles of the patients and the viral CTL epitopes that should theoretically be presented, we found variability of the latter in the archived proviral DNA. This raised the question of the efficiency of curative vaccines based on generic recombinant viruses or viral peptides [17]–[18]

In these patients the virus had sufficient time to accomplish an antigenic drift so the variability of the CTL epitopes at the time of ART success and curative vaccination is not surprising. In the present study, the patients were recruited and followed up not long after their primary infection. The results confirm that it is not possible to predict fully which epitopes will be recognized by the HLA alleles of patients if reference sequences and epitopes are taken as a basis for simulation. Variability of epitopes may be observed in circulating RNA and proviral DNA. For example, p17 74–82 of GOL 040 was poorly recognized throughout the follow-up whereas it should have been easily presented. p17 20–28 of ACT 95387 was presented till month 5.5 but was no longer recognized at months 17.5 and 29.5. We have few samples of archived viral DNA at ART success although they are very interesting for a vaccinal curative approach. In fact, the emerging virus at treatment interruption originates from the proviral DNA so it is crucial to check whether the vaccine epitopes can match with the archived epitopes on the basis of the HLA groove. An interesting example is epitope RT 118–127 in proviral DNA at 16 months post-infection in patient HTM 399 who was under successful treatment and in whom a double population of variants was observed, one of which was not recognized by the HLA groove and accounted for 27% of the total viral population. Therefore, it is not possible to predict fully which CTL epitopes have been finally archived so this finding, together with the potential affinity for the HLA groove, is to be taken into consideration in the quest for a curative vaccine. Nevertheless, we were surprised by the overall low variability of the epitopes in the patients evaluated in this study, who were identified during primary infection and whose points of study had mostly been obtained before treatment. Another surprise is that epitopes varied less than expected in those patients who did not succeed in controlling fully their viral replication. In an animal model, it was recently shown [19] that CD8 T lymphocytes cross-recognize CTL epitopes of mutant SIV sequences but fail to contain the early evolution of escape mutations. Of course, viral production (and viral load) reach high levels during primary infection and immunological control is probably difficult to achieve at this time.

We therefore raise the hypothesis of a lower variability of CTL epitopes in patients close to primary infection versus patients at distance from this event. Our series establishes a trend in favor of this hypothesis but not a definitive conclusion.

We still believe that a vaccine could be a solution in the search for an HIV cure. Promising results have been obtained in a non-human primate model where vaccine-induced CD8+ T cells controlled SIV replication [20]. Both our initial and present studies show that CTL epitopes can exhibit variability compared to the HIV-1 reference, particularly at point(s) of archived DNA that are to be considered for curative vaccination. The limitations of these studies are the low number of patients included and the fact that only circulating blood cells were analyzed, whereas it would be interesting to investigate other compartments, e.g. GALT [21]. Another limitation is the sequencing technique which refers only to Gag and Nef regions by Sanger and RT by UDPS, whereas full genome sequencing by next generation sequencing (potentially UDPS 454) would be more informative. It may be that only a customized vaccine will prove efficient in a vaccinal strategy, given the variability of the epitopes and HLA groove affinity. Nevertheless, crucial questions are raised. Might there be variants for certain epitopes that can be observed only by NGS? Are these variants the same in the different reservoirs and particularly the circulating cells, lymph nodes and GALT? Are the detected variants associated with competent replication viruses?

In conclusion, the Provir/Latitude 45 project aims to study the variability of CTL epitopes in archived proviral DNA of patients at success of first-line ART and who could benefit from a curative vaccine. The initial data show that it is not possible to predict fully which CTL epitopes should be used for vaccination: first because they are variable compared to the reference and also during the follow-up in individual patients; and second because the HLA alleles of the patients are different when CTL epitopes, which themselves are variable, have to be efficiently presented. Therefore there are two thresholds in vaccine efficiency that might not have been crossed in the present trials. In the quest for a customized vaccine, which will be considerably more complex than the vaccines tried so far, it remains to be sure that the CTL epitopes that are efficiently presented are common to the different compartments and that subspecies at the corresponding points do not hamper the homogeneity of the epitopes.

## Methods

### Study patients

Six HIV-1 infected patients from the Canadian primary infection cohort of Montreal were retrospectively enrolled into the Provir/Latitude 45 study. The date of virus infection had been appreciated taking into account the clinical signs, the last negative serological assay and the detuned assay; it was considered to be 14 days before first clinical signs. Written informed consent for medical research had been obtained from each participant and the medical board of Montreal agreed to cooperate with the Latitude 45 study which had received authorization from the “Comité de protection des personnes du Sud Ouest” (DC 2012/48). Viral load and T CD4 lymphocytes were quantitated. Plasma and PBMC samples were frozen at −80°C before being sent to Bordeaux for extraction of nucleic acids and viral sequencing and HLA characterization.

### RNA and DNA extraction

DNA and RNA were extracted as described in the previous Provir study [16].

### PCR amplification of Gag, Nef and Pol regions

Epitopic regions of Gag and Nef were amplified from RNA and/or DNA extracted previously by using primers already described [16] and AmpliTaq Gold with GeneAmp Kit (Applied Biosystem, Foster City, CA). The epitopic region of RT was amplified by using primers of our first study and FastStart HiFi DNApol (Roche).

### Gag and Nef Sanger sequencing

PCR products were sequenced on both strands by using an Applied Biosystems 3500xls Dx Genetic Analyzer and primers from the second PCR. The sequences of the study are available in GenBank under accession numbers: Gag sequences KJ643262–KJ643285 and Nef sequences KJ643286–KJ643308.

### RT Ultra-deep pyrosequencing

RT UDPS was performed by using the Roche GS Junior equipment. Amplicons previously obtained, purified and quantitated were pooled at equimolar concentrations. Clonal amplification on beads (EmPCR) was performed by using the 454 Life Science reagents that enable bidirectional sequencing, composed of 30 cycles of PCR amplification. DNA-containing beads were recovered and UDPS was performed on the GS Junior sequencer (454 Life Sciences; Roche). UDPS generated a median of 5,300 sequence reads per sample. These reads were analyzed with the Amplicon Variant Analyzer software, 454, Roche. The UDPS results of the study are available in GenBank under accession number SRP040731.

### HLA class I typing

Genomic DNA was extracted from frozen white blood cell pellets and quantitated as described above. Intermediate-to-high resolution sequencing was performed by reverse Polymerase Chain Reaction-Sequence Specific Oligonucleotide (PCR-SSO) hybridization by using the LuminexH flow beads LabTypeH assay (InGen, Chilly-Mazarin, France) for the A and B loci. Allelic ambiguities were solved by PCR-Sequence Specific Primer (SSP) amplification by using Olerup assays (BioNoBis, Montfort L'Amaury, France) according to the manufacturer's recommendations. Allele assignment was performed by comparison with official nomenclature and validated by the WHO committee for HLA system factors.

### Immune recognition tools

The viral epitopes in the study were those from the Los Alamos database. Recognition between the HLA groove and the peptides or their variants was predicted by using the immune epitope database (www.immuneepitope.org). We evaluated the affinity of epitopes for the MHC molecules with a MHC IC_50_ (nM) value. Small values are associated with better binders. A value of 500 nM is often used as the threshold between binders and non-binders.
